# Self-Supervised Voxel-Level Representation Rediscovers Subcellular Structures in Volume Electron Microscopy

**DOI:** 10.1109/CVPRW56347.2022.00204

**Published:** 2022-06-19

**Authors:** Hongqing Han, Mariia Dmitrieva, Alexander Sauer, Ka Ho Tam, Jens Rittscher

**Affiliations:** Institute of Biomedical Engineering, Department of Engineering Science, University of Oxford, UK

## Abstract

Making sense of large volumes of biological imaging data without human annotation often relies on unsupervised representation learning. Although efforts have been made to representing cropped-out microscopy images of single cells and single molecules, a more robust and general model that effectively maps every voxel in a whole cell volume onto a latent space is still lacking. Here, we use variational auto-encoder and metric learning to obtain a voxel-level representation, and explore using it for unsupervised segmentation. To our knowledge we are the first to present self-supervised voxel-level representation and subsequent unsupervised segmentation results for a complete cell. We improve upon earlier work by proposing an innovative approach to separate latent space into a semantic subspace and a transformational subspace, and only use the semantic representation for segmentation. We show that in the learned semantic representation the major subcellular components are visually distinguishable and the semantic subspace is more transformation-invariant than another sample latent subspace of equal dimension. For unsupervised segmentation we found that our model manages to automatically rediscover and separate the major classes with errors demonstrating spatial patterns, and further dissect the class not specified by reference segmentation into areas with consistent textures. Our segmentation outperforms a baseline by a large margin.

## 1 Introduction

Biologists use imaging, e.g. microscopy, to study location, shape, amount, interaction, and dynamics of objects of interest. Modern biological imaging is advancing towards large-scale, high-resolution, and multi-dimension. On the one hand, information from multiple sources, with finer details, and across multiple scales enables biologists to better understand mechanisms behind phenomena by observing. At the same time it poses a challenge to extracting hidden insights from this vast and ever-growing amount of low-level signals.

Representation learning [1] has been used in biological imaging to automatically extract features, reduce dimensionality and characterise variation of microscopy data at various scales, such as single cell phenotypes [17,36], intracellular protein localization [11], and protein structure [2]. However, these studies are all limited to learning representations of objects using images completely containing the objects of interest. Learning a representation for all voxels in whole volumes using densely sampled image patches at subcellular level has rarely been done.

In recent years, Focused Ion Beam Scanning Electron Microscopes (FIB-SEM) are producing 3D large-scale near-isotropic nanometer-resolution data, which provides views of whole-cells with clear sub-organelle details [37]. This opens up a unique opportunity for learning a voxel-level (semantic) representation of intracellular architecture. A study like this would give us a holistic and unbiased view into the complete data distribution of appearances, forms, textures or visual motifs of cellular internal organization, without a focus on frequently studied objects. The learned representation could subsequently be clustered to achieve unsupervised segmentation, which could serve as a starting point for human-in-the-loop studies to achieve more precise segmentation or discover spatial distribution patterns of subcellular components.

Lately, learning a voxel-level latent representation for biological imaging data has been done in CIHS (capturing implicit hierarchical structure) [9] by sampling a small image patch around voxel of interest. The authors use Variational Auto-Encoder (VAE) [13, 24] to learn a latent representation, which is subsequently clustered to achieve unsupervised segmentation. The authors of CIHS additionally used triplet loss [3,27] and hyperbolic geometry [18] to achieve an implicit hierarchical structure in which they believe biomedical volumes are organized in the latent space. However, this work has only demonstrated quantitative results on a synthetic dataset and a brain-tumor dataset. They described testing their algorithm on whole-cell cryoET volumes, but only presented qualitative unsupervised segmentation result demonstrating one mitochondrion.

In this work, we learn a largely transformation-invariant representation of small 3D image patches, and use it for unsupervised segmentation of whole-cell high-resolution isotropic volumes. We first train a convolutional VAE using ELBO loss and metric learning [8,15]. At inference time we use the trained encoder to map unseen volumes onto a latent sub-space. Finally, we cluster the latent representation to achieve unsupervised segmentation. We only sample small image patches for training and inference to take advantage of lower data variation and higher data abundance at small scale. We use data augmentation and metric learning to pull semantically similar data together in a designated “semantic” latent subspace. As a result, major semantic classes are visually distinguishable in the designated “semantic” latent subspace, which is more transformation-invariant than another sample subspace with equal dimensionality. Clustering this representation achieved unsupervised segmentation where the three major categories, nuclei, granules and mitochondria, are well separated, and none of them are divided into multiple clusters. Voxels categorized as unrecognized in Reference Segmentation (RS) but mistakenly clustered with nuclei and mitochondria demonstrate spatial patterns visibly different from nuclei and mitochondria. They, together with some other clusters, further dissect the unrecognized RS class (which is unspecified cytoplasmic regions) into components with distinct texture. We adapted and compared to a baseline [9] across different settings. Our model consistently performs better on the task of unsupervised segmentation.

## 2 Related Work

### 2.1 Learning a representation of image patches using VAE and metric learning for unsupervised segmentation

Our approach is inspired by CIHS [9] in that they both use VAE as the base model and apply metric learning to reorganize the latent space. They differ in a number of aspects. The authors of CIHS interpret biomedical imaging data as implicit hierarchical structures, and organized their pipeline around this view by using hyperbolic VAE [18] and multi-scale sampling. We instead observe that texture and local visual features alone are already largely sufficient for telling apart some subcellular structures, while large patches demonstrate too prominent variability for existing data to completely cover. So we only sample small patches for training and inference. We also conduct much more aggressive data augmentation with a wider range of transformations. In addition, we adopt a different metric learning method to more efficiently utilize samples. In the end, we use the metric learning loss to encourage original patches and transformed versions of themselves to be close to each other only in a latent sub-space. This way the representation is disentangled into semantic and transformation subspaces, with the semantic subspace being largely transformation-invariant.

### 2.2 Unsupervised segmentation by clustering image patches

We think of the task of unsupervised segmentation or unsupervised voxel classification as voxel clustering. To cluster the voxels, we actually (1) sample an image patch centered at each voxel of interest, (2) put the image patches into a neural network module to acquire vector representations, and (3) cluster the vector representations. This approach is preferable when global information in images is not necessary for the success of the task. Examples following this paradigm include: JULE (lung cancer micro-CT) [19], CAE (hyperspectral) [21], and IIC (satelite) [10]. These works chose either auto-encoders or convolutional neural networks for representation learning. Compared to these models, the benefits of using VAEs are (1) the representation retains enough information about the overall appearance of the image patch, (2) the representation is a coherent and meaningful distribution of observations in a transformed space, and (3) because of (2) once trained the model can be applied to infer unseen data and generate new data.

### 2.3 Representation learning with VAE and metric Learning

Clustering image patches for unsupervised segmentation only works if representations of patches are distributed in the latent space in a desirable way: representations of the same semantic category are closer to each other than to others, and ideally even form tight clusters. Without supervision, a good way of enforcing this property is to use metric learning [8, 15], such as triplet [3, 27], n-pair [30], lifted embedding [31], batch hard / batch all [6], multi-similarity [35]. Metric learning approaches pull anchor patches closer to positive samples (usually transformed version of anchors) and push them away from negative samples (usually randomly selected samples) simultaneously, in the representation space.

The idea of jointly training a VAE and triplet loss was first explored by [12]. In this pioneering work, the joint model outperforms both metric learning and generative models alone. It divides the latent space into compartments in order to utilize external information of different aspects. Another recent modification to VAE is to maximize the consistency between latent representations of original and transformed versions of the same sample (CR-VAE) [29]. This is achieved by minimizing mutual information between original and transformed samples together with ELBO loss. This variant of VAE has more transformation-invariant latent representations. It is very similar to metric learning but does not use any negative samples. CIHS [9] first applied the idea of combining a VAE and metric learning on unsupervised segmentation.

### 2.4 Disentangling the latent space

In representation learning [1], a desirable characteristic of learned representations is the disentanglement of variable factors in the latent dimensions. Following this principle, a series of generative models are designed with the ability to separate certain variables, such as location, scale, angle, and brightness [4, 5, 7, 16, 38]. This property is highly practical in the field of structural biology, because in protein images, the protein molecule appears in various locations and orientations. For these images, VAE has been used to explicitly separate the variables of rotation and translation, making inference more invariant to these transformations and more focused on semantic category, while enabling more control-lable image generation [2, 25, 39].

## 3 Methods

### 3.1 Learning a voxel-level representation with image patches, VAE and metric learning

Inspired by [9], we learn a voxel-level representation of 3D microscopy volumes. Clustering this representation assigns categories to each voxel, and achieves unsupervised segmentation. In practice, because each voxel is only one scalar, we follow the convention of existing work, and accompany it with surrounding voxels. So effectively we use image patches to represent their center voxels. We use VAE to learn the representation, and additionally apply metric learning loss to make the representation more invariant to common geometric and color transformations.

**Figure 1:**
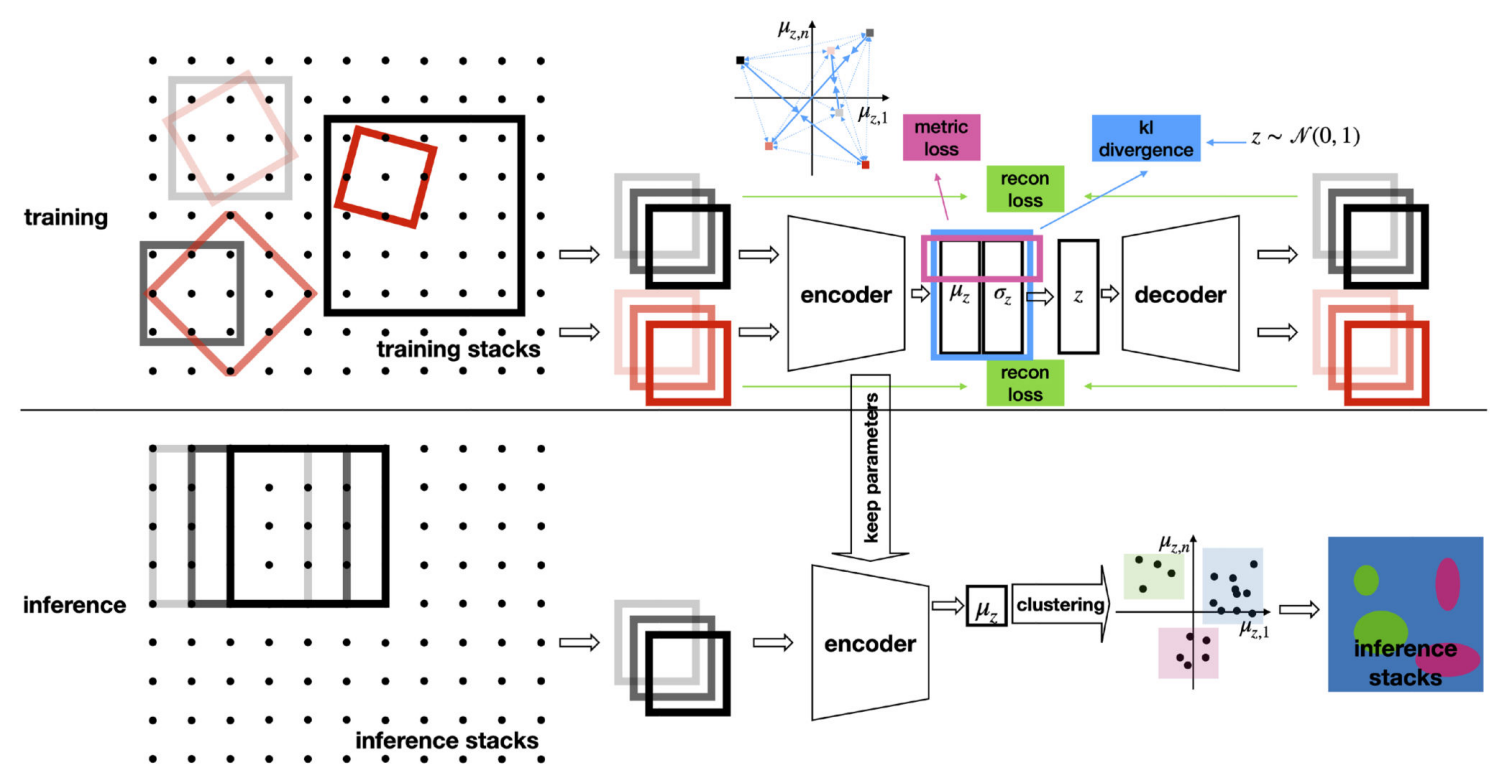
The overall pipeline for self-supervised representation learning and unsupervised segmentation. Training and inference phases are shown in the upper and lower panels respectively. 3D stacks and patches are illustrated as 2D dot grids and squares. Anchor and positive patches are represented as black and red squares.

At training time (Figure 1 top row), anchor and positive patches are sampled using PyTorch grid sample function with fixed grid dimensions. For each anchor there are multiple positive patches, whose centers are located within a small spherical neighbourhood around the center of the anchor. The stack, location and size of anchors are randomly chosen following certain distributions, and positive grids are further allowed to freely rotate and deform. Anchor and positive patches are then augmented with geometric and color transformations. Both anchor and positive patches go through forward pass of a convolutional VAE.

Like a standard ELBO loss, the objective function includes a reconstruction loss between input x and reconstructed input *µ*_x_, and a weighted KL-divergence between standard normal and latent variable z [7, 13, 24]. Note that we normalize the reconstruction term with batch size *b* and volume of patch or sampling grid *a*^3^, and normalize the KL-divergence term with *b* and latent dimension *d* such that

(1) $$L_{recon\;}=\frac{1}{ba^{3}}\text{||x}-\mu_{\text{x}}{\text{||}}^{2},$$

(2) $$L_{KL}=\frac{1}{bd}D_{KL}(N(0,1),N\left(\mu_{z},\sigma_{z}^{2}\right)).$$

Additionally, we use multi-similarity loss [35] to force the representation of anchor patches to move closer to the representations of its own positive patches and farther away from all other anchor and positive patches. The multi-similarity loss is only applied upon the first *d*_*MS*_ dimensions of latent mean *µ*_z_. The multi-similarity term is normalized by batch size (number of samples) and the total number of anchor and positive replicates within each sample (1 + *n*_*pos*_) so that

(3) $$\begin{array}{l} L_{MS}=\frac{1}{b\left(1+n_{pos\;}\right)}\sum_{i=1}^{b}\sum_{j=1\;}^{1+n_{pos\;}}\{\\ \;\frac{1}{\alpha }\log\left[1+\underset{k\neq j}{\sum }\exp(-\alpha (S[j,i,k,i]-\lambda ))\right]\\ +\frac{1}{\beta }\log\left[1+\sum_{k=1}^{1+n_{pos\;}}\underset{l\neq i}{\sum }\exp(\beta (S[j,i,k,l]-\lambda ))\right]\}. \end{array}$$

Here *α, β* and *λ* are fixed hyper-parameters. *α* and *β* act as scales and *λ* is a margin. *S* is a pre-computed tensor recording distances between all *b* samples and all 1 + *n*_*pos*_ replicates, which means all *b* × (1 + *n*_*pos*_) anchors and positives within a batch. *S*[*j, i, k, l*] is the distance between *µ*_z_ of sample *i* replicate *j* and sample *l* replicate *k*, using first *d*_*MS*_ latent dimensions. The final batch loss is a weighted sum of all the loss terms, as

(4) $$L_{batch\;}=L_{recon\;}+\theta_{KL}L_{KL}+\theta_{MS}L_{MS}.$$

At inference time, we sample patches centered on each voxel of the inference stacks, with fixed orientation and size (Figure 1 bottom). Patches are fed into the encoder of the VAE, and the semantic dimensions of the latent mean (*µ*_z_) are kept for clustering.

Clustering is conducted using Mini-Batch K-Means (MBKM) implemented in scikit-learn [22, 28]. We chose MBKM due to its scalability and performance. Each validation or test volume has approximately 0.5 billion vectors and the representation has 8 dimensions. For such large amount of data, most other clustering methods require sampling and performed worse than MBKM. Mini-Batch GMM based on PyMC [26] works without sampling but did not achieve as good results as MBKM. After clustering, unsupervised segmentation is completed.

### 3.2 Exploiting local and textural stereotypicality by keeping patches small

**Figure 2:**
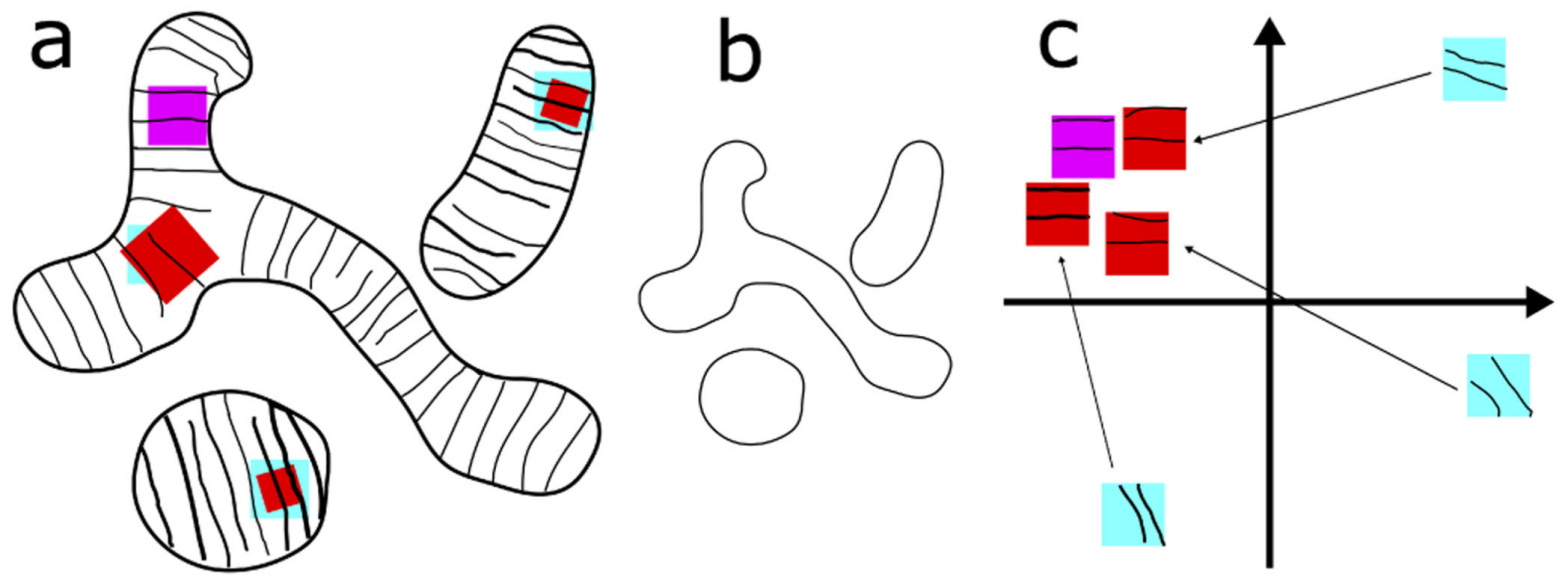
Subcellular structures have plastic shapes but stereotypical texture. We take mitochondria as an example. (a) In many cell types and under some imaging modalities, mitochondria have repetitive parallel stripes or edges perpendicular to the direction of their extension. They look stereotypical locally (the visual patterns in magenta and red patches are similar). (b) The shape of mitochondria is very flexible overall but this does not affect the statement we made in (a). (c) Latent representation of patches. Latent representation of red and magenta patches are already close because they are similar in data space. Arrows: metric learning pulls representations of cyan patches close to red and therefore magenta. If this succeeds, representations of patches with stripes form a cluster, regardless of orientation and scale. Clustering this representation achieves unsupervised segmentation of mitochondria. The scheme is illustrated in 2D for convenience.

The scale and shape of subcellular structures (e.g. mitochondria) are sometimes rather diverse and flexible (Figure 2 b). But at a smaller scale, the texture and local visual features are more stereotypical or consistent across the entire object and among different objects (Figure 2 a). In other words, small patches within objects of the same class demonstrate self-similarity after geometric and color transformations (Figure 2: cyan patches are similar to magenta patch after transformed into red patches).

Based on this observation, we speculate that sampling small patches for voxel-level representation learning has several benefits. First, the dimension of data (image patches) decreases. Further, because the appearance of data (image patches) is less variable at a smaller scale, they can be represented by an even lower-dimensional latent space. Lastly, data become more abundant. Since we have much more data to fill in a much smaller representation space, examples / observations become more dense for the latent distribution, and the quality of the learned representation should be much better.

Another benefit of using small patches is the reduction in the amount of patches involving boundaries of subcellular structures. This strengthens the argument that we can use patches to represent voxels.

### 3.3 Data augmentation and metric learning for a transformation-invariant representation

Our goal is to acquire a representation where semantically similar patches (Figure 2 magenta and cyan patches) are close to each other and separated from other patches (not illustrated). If this is achieved, simply clustering the representation will achieve unsupervised segmentation.

However, at training time, it is not possible to correctly identify positive pairs like cyan and magenta patches, without supervision. Fortunately, since magenta and red patches already look similar, it is reasonable to believe that their latent representations will also be close. Now we only need the representations of cyan patches to be close to those of red patches (transformed versions of cyan patches themselves). We use metric learning to achieve this goal.

However, if semantically similar patches with very different appearances (e.g. orientations and scale) are very close in the representation space, information will be lost and reconstruction will be affected. Here, metric learning only act on a latent subspace. In this way we encourage this subspace to become transformation-invariant, and the rest of the latent space to facilitate reconstruction.

## 4 Experiments

### 4.1 Data

OpenOrganelle is an open online repository for high-resolution cell and tissue imaging data with different specimen types and imaging conditions [37]. Among these datasets, we choose to test our method with a Focused Ion Beam Scanning Electron Microscopy (FIB-SEM) dataset of primary mouse pancreatic islets *β* cells “BetaSeg” [20], because mitochondria in this dataset have characteristic texture. We downloaded preprocessed data following a link provided by their publication. Preprocessing includes cropping cells out of whole tissue stacks and binning voxels, lowering resolution from 4nm to 16nm.

The authors treated isolated pancreatic islets with either high or low dosage of glucose. For each treatment group, a large 3D volume containing multiple cells is acquired. Subsequently they cut both volumes into smaller stacks, each fully containing one cell. Eventually the data consists of a low-glucose group of 3 stacks (cells) and a high-glucose group of 4 stacks (cells).

The dataset contains binary segmentation masks of 7 categories (centrioles, nucleus, membrane, microtubules, golgi, granules, mitochondria) generated by manual annotation or supervised segmentation (with or without manual curation). Some of these binary masks overlap. We use these masks as RS after eliminating the overlaps between the binary masks following the principle that manual annotation / curation should prevail. After preprocessing we acquire in total 8 classes (the 7 plus the rest which we name “unrecognized”). Out of the 8 classes, 4 (golgi, membrane, microtubules, centrioles) are much smaller than the other 4 (nuclei, granules, mitochondria, unrecognized).

We only use the high dosage group for our experiments because the stacks of low dosage group have low contrast which results in loss of signal. We hold “cell 4” (*d* × *h* × *w* = 1022 × 545 × 1082) out for testing, and use “cell 1” (1097 × 699 × 760), “cell 2” (1043 × 606 × 870), “cell 3” (1023 × 676 × 845) for 3-fold cross-validation. In each round of cross-validation we train with 2 cells (stacks) and infer with 1 cell (stack). At testing we train with cells 1, 2, 3 and infer with “cell 4”. During both cross-validation and testing we trained for different lengths (2 or 3 million anchors per stack). For comparison we trained CIHS with 10 million anchors per stack.

### 4.2 Hyperparameters

The sampling grid for anchor and positive patches have 16 points in each dimension. Their physical sizes vary from 4 to 12 at training and are fixed to be 8 at inference. This is equivalent to resizing sub-volumes of sizes 5 to 13 (training) and 9 (inference) into patches of size 16. At training phase, each batch has 128 anchors and each anchor is accompanied by 4 positives. We learn a 64-dimensional latent representation, out of which the first 8 receive self-supervision signal from metric learning.

### 4.3 Representation

We use t-SNE [23, 32, 33] to visualize two latent subspaces: dimension 1 to 8 (with self-supervision signal) and 9 to 16 (without self-supervision signal).

**Figure 3:**
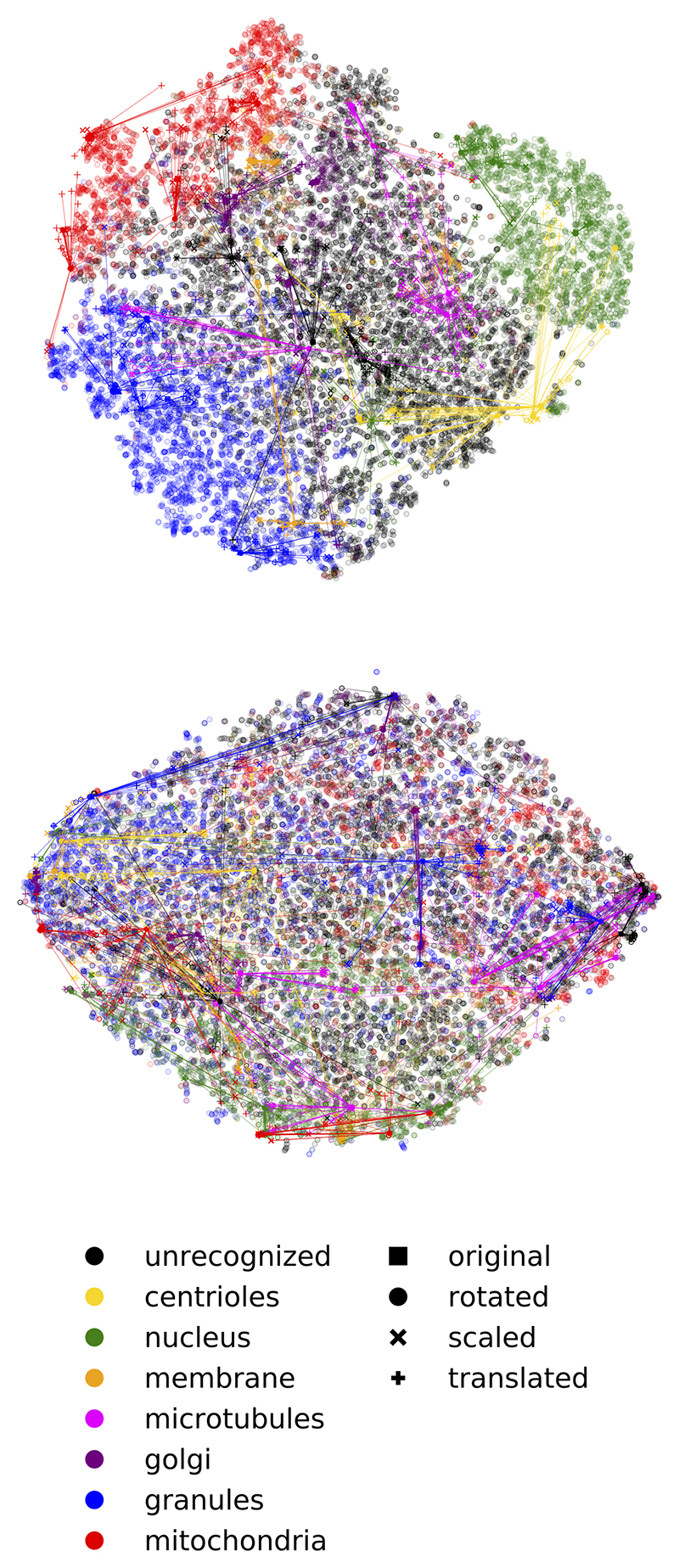
Semantic subspace demonstrates better separation of semantic classes and better transformation-invariance. Representation of cell 4 (test stack) is shown. Dimensionality reduction is conducted using t-SNE. Top: latent dimensions 1 to 8. Bottom: latent dimensions 9 to 16. Transparent markers: patches proportionally sampled from each RS class and used to train t-SNE. Opaque markers: patches uniformly sampled from each RS class (original and transformed patches are connected by lines).

We sample the latent representation of test volume from each RS class proportional to the sizes of the classes, then use two subspaces, dimensions (dim) 1 to 8 and 9 to 16, to fit 2 t-SNE models, and visualize them with 2 scatter plots (transparent dots in Figure 3). Each RS class is marked by a different color. As can be seen from Figure 3, the 3 major RS categories are visually separable in the semantic subspace (dim 1 to 8, upper panel), but not in another latent subspace (dim 9 to 16, lower panel).

We sampled from each RS class equally 4 patches and transform them with rotation, scaling and translation. We infer the original and transformed patches and acquire their representations in the two subspaces (Figure 3 opaque dots). We find that the semantic subspace is more invariant to transformations, as the representations of transformed patches are closer to the representations of original patches, for the larger classes (unrecognized, nucleus, granules, mitochondria). Since dim 9-16 are not trained differently than dim 17-64, we speculate that dim 17-64 should also be less transformation-invariant than dim 1 to 8.

### 4.4 Evaluating unsupervised segmentations

Following the practice of [9], we evaluate unsupervised segmentation with Dice Similarity Coefficient (*F*_1_-score) and Hungarian algorithm [14]. First, we calculate a confusion matrix $C_{n_{Pr}\times n_{RS}}$ by comparing RS and model prediction. Here *n*_*RS*_ = 4 and 4 ≤ *n*_*Pr*_ ≤ 10. Then, a Dice matrix **D** of the shape as the confusion matrix is computed: $D_{ij}=\frac{2\times C_{ij}}{\sum_{k=1}^{n_{RS}}C_{ik}+\sum_{l=1}^{n_{Pr}}C_{lj}}$. We use the SciPy linear_sum_assignment function to find the minimum weight match of the cost matrix −**D** [34]. The number of entries in this match is *n*_*M*_ = min(*n*_*RS*_, *n*_*Pr*_) = 4. The final Dice score is the mean of **D** entries in this match.

### 4.5 Unsupervised segmentation

**Figure 4:**
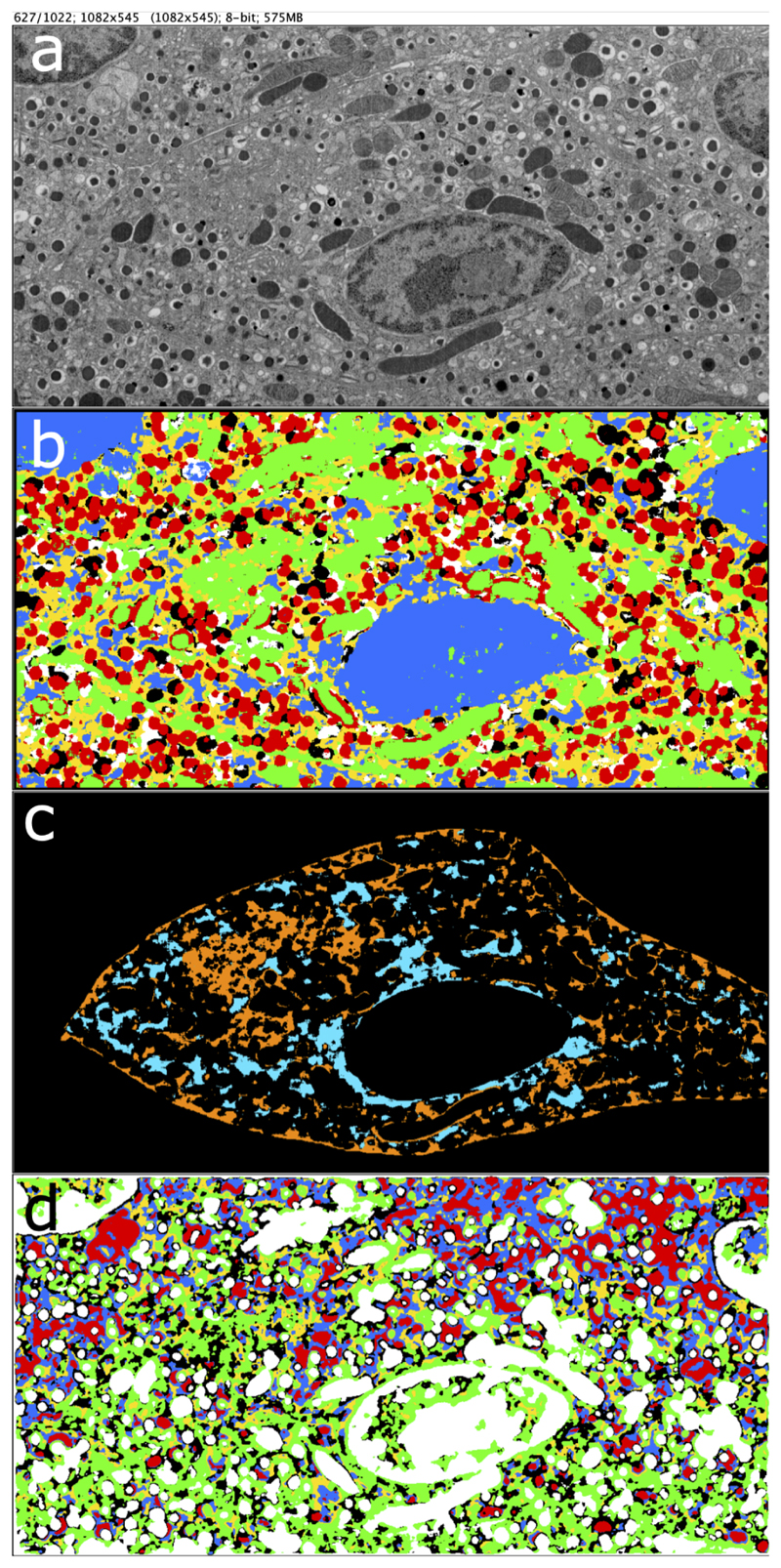
Example of our unsupervised segmentation. (a) Raw stack (section 627 of cell 4). The large oval rough structure with bright and dark regions are nuclei. The cylinder shaped bright or dark structures with stripes are mitochondria. The small round dark structures wrapped by brighter surface are granules. (b) Qualitative segmentation result using K-Means (*k* = 6). The color legend can be found in the right of Figure 5 b. Each color (segmentation class) corresponds to a row in Figure 5 a and b. (c) Errors. Orange is voxels clustered with mitochondria but are not. Cyan is voxels clustered with nuclei but are not. (d) Qualitative segmentation result of CIHS also with *k* = 6 for hyperbolic K-Means.

**Figure 5:**
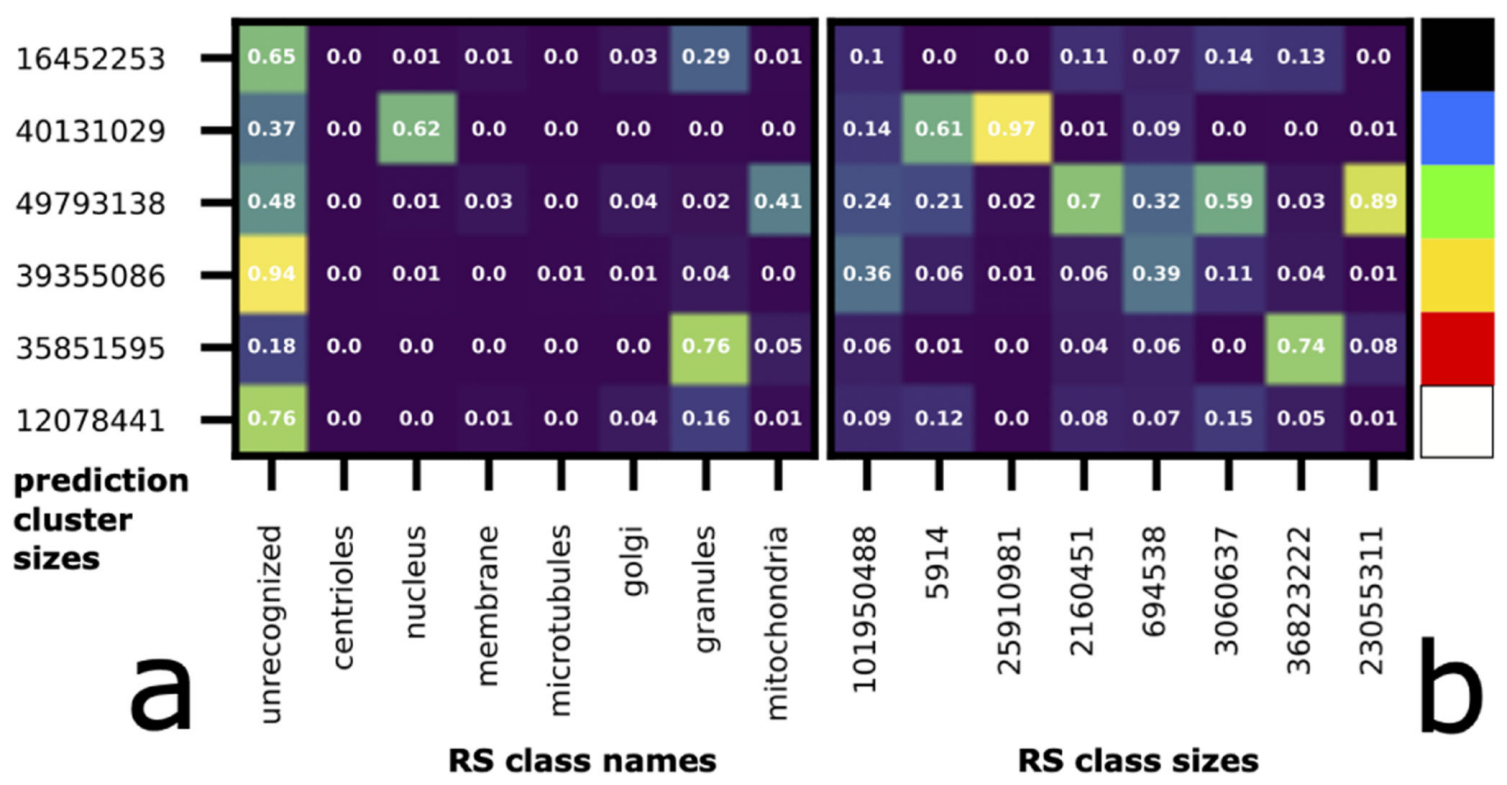
Quantitative unsupervised segmentation result shown as confusion matrices corresponding to Figure 4 b and c. Each row is a prediction class / cluster; each column is a RS class. X labels: names (in a) and sizes (in b) of RS classes. Y labels: sizes of prediction classes / clusters. Confusion matrix in (a) is normalized within row and the one in (b) is normalized within column. Correspondence between confusion matrices rows and colors in Figure 4 b (there is also legend to the right of the matrices): from 1 to 6 are black (mostly unrecognized and partially granules), blue (mostly nuclei and partially unrecognized), green (mitochondria and unrecognized), yellow (unrecognized), red (granule), white (unrecognized). Correspondence between confusion matrices entries and colors in Figure 4 c: cyan is row 2 column 1; orange is row 3 column 1.

First we demonstrate that our method is able to automatically rediscover subcellular structures without any annotation. Cell 4 (test stack) is segmented by a model trained with 2 million anchors each from cells 1, 2, 3 (Figures 4, 5).

As can be seen from the segmentation masks, the three major classes nucleus, granules and mitochondria are well separated with each other, as each of the three major RS classes are covered by a separate mask (nucleus: blue, granules: red, mitochondria: green, Figure 4 b). This is also evidenced quantitatively in Figure 5 b, where each of these RS classes overlap almost exclusively with a separate prediction class/row (nucleus / col 3: row 2, granules / col 7: row 5, mitochondria / col 8: row 3). We further examine how much these prediction classes overlap with other RS classes (Figure 5 a rows 2, 3, 5). Mitochondria (col 8, row 3) and nucleus (col 3, row 2) each are clustered with some voxels in the unrecognized class (col 1, row 3 and col 1, row 2), but these error areas demonstrate spatial patterns that are visibly different from mitochondria and nuclei respectively (col 1, row 3: orange in Figure 4 c; col 1, row 2: cyan). Specifically, much of the orange area are located near cell membrane, and also form a network-like clump to the top-left of nucleus; much of cyan are near and connected to the nucleus. We find out that both membrane and Golgi are mostly clustered in prediction class 3, together with mitochondria. This can be explained by the fact that they look similar (with stripes/edges) at a very small scale.

Interestingly, our algorithm automatically dissects the unrecognized class (Figure 5 col 1) of RS into several categories with distinct textures (Figure 4 a, b, c). Row 1 (black in b): smooth bright areas around granules. Row 2 (cyan in c): rough dotted areas e.g. around nuclei. Row 3 (orange in c): areas with stripes/edges like mitochondria, e.g. area near membrane but in unrecognized category in RS. Row 4 (yellow in b): the major unrecognized class. Row 6 (white in b): rather bright area with sparse dots and edges.

During cross validation, none of the clusters would be assigned exclusively to any of the four smaller classes, no matter which *k* we choose. This could be explained by the fact that none of the clusters is small enough to only include one minor class. So now we only quantitatively evaluate with four RS classes, namely nucleus, granule, mitochondria, and unrecognized (which now includes the previous unrecognized, centriole, microtubule, membrane and golgi). We compared our approach with CIHS [9] using multiple *k* values for KMeans clustering and different training lengths for our model. Our model consistently performs better in cross-validations and test (see Table 1). Figure 4 d demonstrates segmentation by CIHS. Mitochondria, granules and parts of nucleus are not successfully separated.

### 4.6 Efficiency

On a computational node with 24 CPU cores (2.30GHz) and 4 GPUs (GeForce RTX 2080 Ti; only one GPU used), training takes 4h per million anchors for our method and 0.5h per million anchors for CIHS; inference (0.5 billion voxels) takes 24h with our method and 4h with CIHS.

## 5 Conclusion and discussions

In this work, we introduced a model that learns a voxel-level representation of volume microscopy data, and used it to automatically segment whole-cell intracellular architecture. In our learned representation, major semantic classes are visually distinguishable. Clustering this representation results in a unsupervised segmentation that successfully separates nuclei, granules, and mitochondria, as none of these structures are identified within multiple clusters. Parts of unrecognized class that are mistakenly clustered with nuclei and mitochondria demonstrate spatial patterns that are visually distinguishable with nuclei and mitochondria, respectively. These regions together with other clusters, also demonstrate distinct textural features and further dissect the unrecognized class regions that are not specified by the RS. Overall, this method provides an unbiased view into the textural similarities between different cellular components.

The learned representation and the unsupervised segmentation open up many possibilities. The representation can be readily applicable for weakly supervised segmentation that only requires a small amount of point annotation or scribbles. It also enables interactive inspection across the latent space and data space. For example, biologists could click on a dot in the t-SNE plot and see which voxel it represents. After this, users could even draw polygons and assign class as they wish. The unsupervised segmentation result can facilitate interactive post-processing by allowing users to assign different categories to different connected components of voxels of the same cluster. For example, voxels near cell membrane make up a substantial part of errors for mitochondria cluster. They are visually identifiable as not mitochondria, and possibly not connected to mitochondria. So in an interactive setting users could click and change its class, making segmentation much more precise with a few clicks. Effectively, the representation and unsupervised segmentation transformed a half billion voxel dataset into a scatter plot and a division into a few components, each demonstrating distinct characteristics and some overlap considerably with known semantic classes. Importantly these are done with no human annotation at all.

Although the major categories are not overlapping prominently in the representation space, they are not separated well enough for clustering to work robustly. Given this non-perfect representation, segmentation could change when the length of training or number of clusters varies. And there might be no good *k* values for K-Means to produce a good enough unsupervised segmentation.

An innate problem of the proposed model is that it does not tell apart areas of similar texture but with different shape, such as mitochondria, Golgi apparatus and cell membrane. In the future, we are interested in learning a voxel-level representation that encodes not only a very small neighbourhood around voxel of interest but also appearance at larger scale.

## Figures and Tables

**Table 1:** *F*_1_-score (Dice similarity coefficient) for unsupervised segmentation under 3-fold cross-validations (upper half of table) and test (lower half of table). *x*M/stack means: *x* million anchor patches are sampled from each training stack. In cross-validation and testing models are trained with 2 and 3 stacks respectively.

K	CIHS (10M/stack)	Ours (2M/stack)	Ours (3M/stack)
4	0.363 ± 0.021	0.475 ± 0.016	0.471 ± 0.022
5	0.336 ± 0.060	0.590 ± 0.067	0.593 ± 0.040
6	0.317 ± 0.018	0.579 ± 0.080	0.673 ± 0.003
7	0.324 ± 0.065	0.606 ± 0.054	0.579 ± 0.049
8	0.334 ± 0.019	0.556 ± 0.066	0.534 ± 0.072
9	0.318 ± 0.035	0.564 ± 0.028	0.593 ± 0.017
10	0.305 ± 0.037	0.525 ± 0.030	0.612 ± 0.019
4	0.308	0.625	0.664
5	0.243	0.659	0.623
6	0.291	0.647	0.658
7	0.432	0.643	0.648
8	0.291	0.560	0.632
9	0.342	0.578	0.574
10	0.337	0.567	0.542
